# Combining Listening Cafés and a Games‐Based Co‐Design Approach for Public Involvement With Underserved Communities: A Methodology and Lessons Learned From Health Research

**DOI:** 10.1111/hex.70603

**Published:** 2026-02-17

**Authors:** Sian Holt, Sarah Newman, Julie Powell, Kate Henaghan‐Sykes, Sonia Newman, Miriam Santer, Leanne Morrison

**Affiliations:** ^1^ Primary Care Research Centre University of Southampton Southampton UK; ^2^ Research Support Service (RSS) University of Southampton and Partners (USP) Southampton UK; ^3^ Psychology and Primary Care Research Centre University of Southampton Southampton UK

**Keywords:** co‐design, creative engagement, evaluation, games, health inequalities, patient and public involvement (PPI), underserved communities

## Abstract

**Background:**

Meaningful involvement of underserved communities in health research requires consideration of structural, cultural and linguistic diversity. Games offer promising ways to foster engagement with complex topics and create shared language. However, there is limited evidence on the effectiveness of games for enabling meaningful Public Involvement in health research and minimal methodological guidance on how to facilitate games‐based co‐design with underserved groups. This paper evaluated a combined participatory Listening Café and games‐based approach to public involvement, aimed at supporting meaningful conversations about health with community members, reflecting on the process and lessons learned to establish a replicable methodological model for inclusive public involvement in health research.

**Design:**

We collaborated with community partners from two Family Hubs in Southern England to plan and deliver co‐design sessions. Initial meetings addressed preferred ways of working, event locations, accessibility, ownership of the final product and budgeting. The sessions took place in the community and adopted the Listening Café model, which is a participatory approach for public involvement that builds trust through shared food and informal conversations. The process included three co‐design sessions and a follow‐up 3 months later. Evaluation methods included feedback forms, verbal check‐ins and written reflections from researchers, community partners and community members.

**Results:**

Through a series of Listening Cafés, we co‐designed the card game, ‘Me: Inside and Out’, to encourage conversations about the challenges of living with health conditions with underserved groups. The game facilitated rich, meaningful conversations, fostered empathy and enabled community members to share their lived experiences. Community members reported feeling heard, valued and more connected from being involved in the co‐design process, playing the game and understanding more about each other.

**Conclusion:**

A combined participatory Listening Café and games‐based co‐design approach for public involvement can effectively involve underserved communities in health research. Cultural sensitivity, shared ownership and relationship‐building are crucial processes for fostering inclusion. The Listening Café model proved effective in creating safe, informal spaces for dialogue. Researchers can adopt this methodological approach for public involvement to address perceived barriers to involving underserved communities, co‐producing outcomes that reflect the voices of those it aims to serve.

**Patient or Public Contribution:**

Community partners (Sarah and Julie) supported planning the sessions. Community members attended and contributed to co‐design sessions.

## 1 Introduction

In the UK, the National Institute for Health and Care Research (NIHR) distinguishes between patient and public involvement (PPI) and community engagement and involvement (CEI). PPI refers to research conducted *with* or *by* members of the public, positioning those with lived experience as active collaborators to ensure research is relevant and meaningful [1, 2]. The NIHR provides standards to support high‐quality PPI practice [3, 4]. In contrast, CEI focuses on inclusive participation and building authentic partnerships with people who share common interests or circumstances [5]. It uses participatory approaches to involve communities throughout the research process, ensuring activities are contextually appropriate and effective. While PPI emphasises co‐design and decision‐making, CEI prioritises awareness, dialogue and representation, especially of marginalised groups. Together, PPI and CEI foster more inclusive, transparent and impactful health and social care research.

Meaningful involvement of underserved groups in health research requires careful consideration and planning to overcome barriers such as limited funding, power imbalances, inaccessible language and lack of diversity [6, 7, 8]. Traditional PPI methods (like formal advisory groups) often fail to engage underserved groups due to cultural, educational and linguistic mismatches, as well as issues like researcher mistrust and mental burnout [9, 10, 11]. PPI activities also go unreported, leading to a lack of shared learning within both scientific and community groups [12]. To address this, inclusive approaches must be developed that reflect the needs of underserved groups. Frameworks like NIHR's INCLUDE project and Critical Participatory Action Research (CPAR) offer guidance for adapting research methods to be more democratic, reflective and accessible to groups such as ethnic minorities, people with low literacy and those with complex health needs [13, 14].

Creative and participatory methods are increasingly used in health research to build trust, foster shared ownership and support inclusive dialogue [15, 16, 17, 18]. One effective creative method is the use of games, which create safe, engaging environments for dialogue on sensitive health topics [19, 20, 21, 22, 23, 24, 25, 26, 27]. Playing games can foster emotional connections and rich engagement, making complex information more accessible, offering culturally appropriate experiences for diverse communities [25, 26, 28, 29, 30, 31]. However, public involvement studies that might appear similar to games typically involve interventions or tools, rather than actual games [32]. They also tend to focus on digital or app‐based tools, rather than physical games such as board or card games [33]. Participatory approaches to co‐creating games that are essential for ensuring cultural relevance and relatability are rarely employed, often being perceived as too time‐consuming or technically complex to implement [28, 34].

Overall, there is a lack of evidence for using games to facilitate meaningful public involvement with underserved groups. This paper aims to evaluate the use of participatory Listening Cafés and a games co‐design approach for public involvement to facilitate meaningful conversations about health with underserved communities, and to reflect on the process and lessons learned. Where possible, we share reflections from our community members and community partners in their own words. This study offers one of the first UK examples of combining the participatory Listening Café methodology with physical games‑based co‑design as a PPIE approach, rather than as a clinical or digital intervention, co‑creating the conversation card game ‘Me: Inside and Out’ with underserved communities. By grounding the process in trust, cultural sensitivity and shared ownership, we contribute a practical, replicable model for inclusive public involvement in health research.

We aim to answer:

- Does the combination of participatory Listening Cafés and games‐based co‐design approach lead to effective public involvement?
- What are the key methodological and practical considerations when planning future Listening Café events with community partners and co‐designing games with community members?

## 2 Methods

This methods section follows the GRIPP‐2 reporting guidelines for PPIE research, see Appendix A [35].

### 2.1 Context

Our research team were conducting semi‐structured interviews to examine social care needs and inform the development of an intervention to support people who are living with multiple long‐term health conditions (MLTCs) with their daily non‐medical challenges [36, 37]. Throughout recruitment for the interviews, we noted a lack of included voices from ethnically and socially diverse groups, despite targeted recruitment strategies (e.g., reaching out to specific charities). We aimed to supplement the research with a creative approach to engagement to ensure the needs of these underserved groups were addressed within the intervention design.

### 2.2 Design

The Listening Café model was used to engage underserved communities in the development of a conversation‐based game. The Listening Café model was originally developed by Sonia Newman and Kate Henaghan‐Sykes as part of The Finding Out Together team at the University of Southampton in collaboration with members of the Wessex Public Involvement Network and further developed in partnership with Family Hub [38]. It has since been a successful approach to facilitate and support researchers to engage with and involve people from underserved communities in research. The principle of the Listening Café is that the best conversations happen around food within safe and trusted places. Listening Cafés take place in existing community spaces, working closely with community partners and existing groups. Details of the project are shared with expressions of interest from community members who wish to take part. This contrasts with traditional research, where people are invited directly to take part, with researchers being invited to attend rather than researchers inviting the public to get involved. To help develop ongoing and reciprocal relationships, Listening Cafés meet for three to 4 weeks to focus on a particular research topic. At a later date, a follow‐up session is delivered to explore how the community input has influenced the research. Each 2‐h session focuses on building trust through sharing food, creative activities and informal conversations to explore what matters most to the community.

### 2.3 Theoretical Underpinning

This project is grounded in the principles of co‐production and participatory design, which emphasise shared power, mutual respect and collaborative knowledge generation between researchers and the public [39, 40, 41, 42]. The co‐design approach used in this project aligns with the NIHR INVOLVE definition of public involvement, where research is conducted with or by members of the public rather than to, about, or for them [1]. The Listening Café model draws on relational and dialogic theories of engagement, which posit that trust and shared understanding are built through informal, reciprocal interactions [43]. These theoretical foundations informed the structure of the sessions, the facilitation style and the emphasis on creating a safe, inclusive environment for meaningful dialogue.

### 2.4 Community Partners and Community Members

Our community partners (Sarah and Julie) from two Family Hubs in Southern England collaborated with us, helping to support and connect us with community members. Family Hubs are local community organisations that provide support for families and young people aged 0–19 living in disadvantaged areas, providing play sessions, healthcare support, employment support and more.

Community members were parents and staff from underserved groups, including diverse ethnic and socioeconomic backgrounds.

### 2.5 Planning and Conducting PPIE Events

#### 2.5.1 Planning Meetings

Sonia Newman and Kate Henaghan‐Sykes connected Sian Holt (lead researcher) with two Family Hubs within areas of higher deprivation in Southern England. Sonia and Kate also continued to play an active role in advising, planning and facilitating the sessions. This helped to make sure our planning and sessions remained relevant and responsive to community members' needs.

Initial planning discussions consisted of four meetings, which later involved our community partners and co‐authors (Sarah Newman and Julie Powell). During planning, we considered:

- Preferred ways of working,
- how we would connect with community members,
- best event location,
- session materials,
- dietary needs,
- accessibility needs,
- ownership of the final co‐created game (including IP stance and power balance),
- budgeting.

Sian engaged in regular self‐reflection and planning to balance how to keep co‐design discussions open and led by community members, whilst still informing the overall design of a game. Sian also recruited an artist (via a series of job interviews) who had the right ethos and understanding of the co‐design process.

#### 2.5.2 Co‐Design Sessions

**Figure 1:**
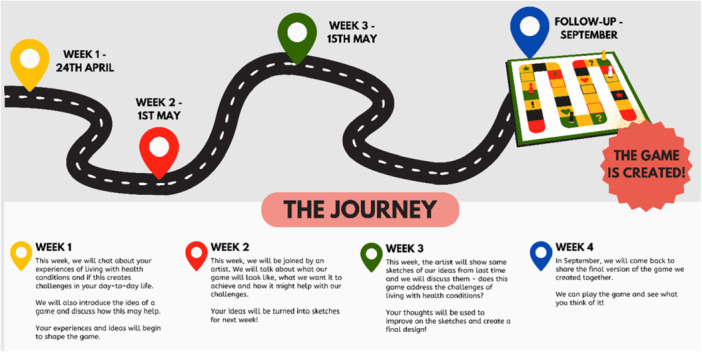
The co‐design journey.

The co‐design process took place over three sessions, with a follow‐up session 3 months later. Figure 1 outlines the co‐design journey, which we shared with our community members to explain weekly progress toward the final co‐creation of our game.

All activities took place in community locations familiar to community members, embedded within existing event structures, for example, pre‐existing Listening Café events or staff meetings. All documents used to prompt discussion or facilitate evaluation were co‐designed with our community partners, with community member needs and accessibility in mind. For example, larger font sizes, materials designed in Plain English. See Appendix B for example documents and the schedule of the planned co‐design process.

### 2.6 Analysis and Evaluation

The analysis and evaluation of the PPI process were informed by:

- Structured feedback forms are completed by community members at the end of each Listening Café session, capturing reflections on engagement, relevance and perceived value. This included Likert scales and free‐text boxes for qualitative responses.
- Verbal check‐ins with community members during sessions to gauge real‐time emotional responses, group dynamics and comfort levels. Observations made during these verbal check‐ins were noted by the lead researcher during and after each session.
- Written reflections by the lead researcher and community partners, documenting observations, group interactions and ideas shared. These reflections were written during and after each planning meeting and session by the lead researcher (Sian), and after project completion for community partners and co‐authors (Sarah and Julie)
- Anonymous contributions during sessions via post‐it notes and paper prompts, allowing community members to share sensitive or personal insights without public disclosure.

Our community partners decided that audio recording the sessions was not appropriate within the community setting, given that this was a PPI activity built around maintaining trust and safety [44]. Recording could have introduced concerns about confidentiality and created a barrier to open, honest dialogue, which is central to meaningful involvement. We focused on evaluating the combined participatory Listening Café and games‐based co‐design approach, referring to the UK Standards for Public Involvement (in particular ‘working together’) [3].

Data from these sources (e.g., changes in Likert scale scoring, free‐text qualitative feedback and written reflections) were integrated and discussed by the lead researcher and wider team. We used an inductive, descriptive approach informed by reflexive thematic analysis to identify recurring ideas and lessons. There were no divergent views to be included.

#### 2.6.1 PPIE

Public Involvement was central to the project. Community partners were involved from the planning stage, helping to shape the format, location, accessibility and content of the sessions. They also co‐facilitated Listening Cafés and contributed to the authorship of this paper (dissemination). The combined participatory Listening Café and games‐based co‐design approach for public involvement enabled community members to shape the game content and structure, ensuring it reflected their lived experiences and priorities.

## 3 Results

### 3.1 Description of the Game

**Figure 2:**
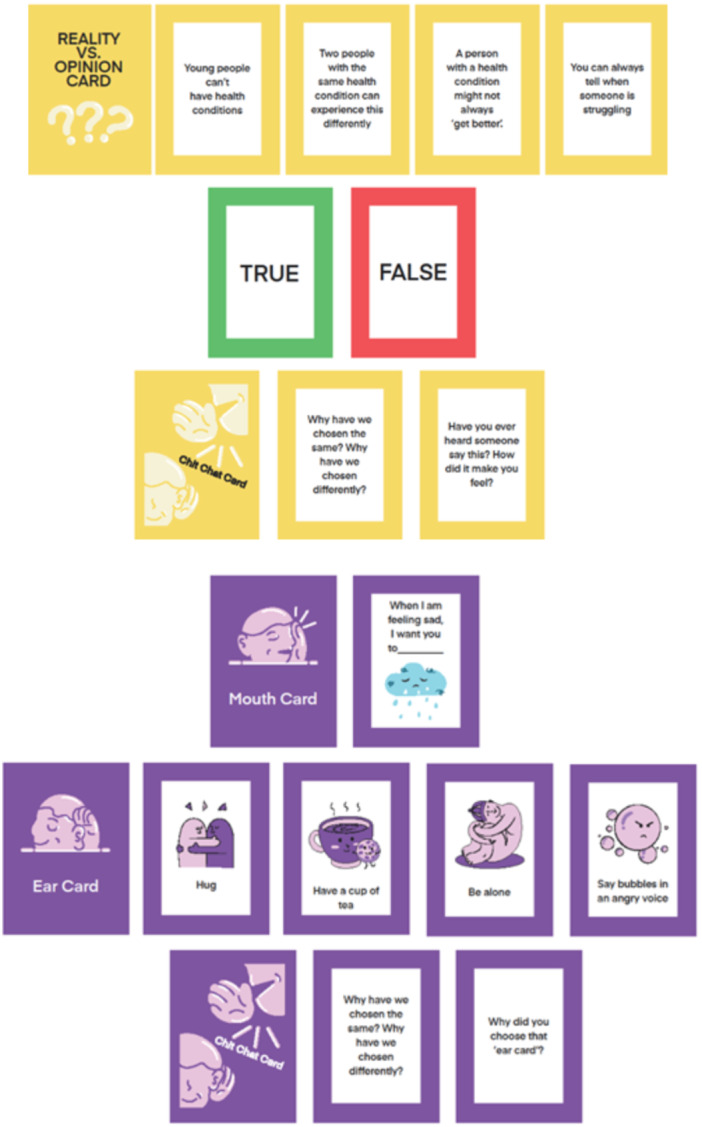
Examples from both the purple pack and the yellow pack of ‘Me: Inside and Out’.

The final co‐designed game was a conversation card game called ‘Me: Inside and Out’. Community members wanted a game to help other people understand them better. They gave ideas for game mechanics such as ‘filling in the blanks’, ‘matching’ and ‘true/false’. During community member discussions, these ideas merged into the final format of the game (conversation card game). The game contained two packs, focusing on priorities identified by community members (Figure 2):

(1) A purple pack which gives space to communicate and align on ways to support each other when feeling sad, angry, tense, frustrated or in pain. This pack came from the idea that our loved ones often do not know the best ways to support us, and these conversations often only happen in times of crisis or high emotion. Having a space for these conversations outside of moments of crisis would allow a sense of shared understanding of what our own personal preferences for support are, and where we might have previously misunderstood the support preferences of other people. This allows players to better understand and support one another.

For this pack, our community members championed the inclusion of humour, reflecting on the fact that conversations and experiences about our health are often negative. They suggested adding ‘action cards’ to break the tension of deep conversations. These action cards also facilitate a sense of ‘togetherness’ by engaging in something silly together, for example, ‘say the word bubbles in an angry voice’. Seeing how these worked in practice was truly revolutionary, and something the researchers alone would not have suggested or felt confident to implement.

(2) A yellow pack which presents statements or common misconceptions about what it's like to live with health conditions. This pack came from the idea that many people do not understand what someone living with health conditions is going through. The statements in this pack are based on things that our community members had heard and experienced. This pack allows space to challenge these misconceptions and discuss how this made them feel, sharing common experiences. This allows players to better understand one another and challenge the way other people think in a safe and fun way.

### 3.2 Experience of the Co‐Design Process

From feedback surveys, community members reported a positive experience from being involved in this co‐design project, with their Likert scale scoring increasing from the first session to the final session on statements related to enjoyment, creation of a safe space, feeling listened to and feeling the sessions were worthwhile. From qualitative feedback from surveys, they enjoyed ‘working together’ and valued the opportunity to ‘speak up about ideas’. They felt that everyone was able to join in and had a voice within sessions. Even quieter group members expressed that they were able to meaningfully contribute their own thoughts and experiences. The group felt a greater sense of understanding of each other from being involved, feeling less ‘alone in the world’ with everyone facing the same issues (‘we are all in the same boat’). They learned through the co‐design process that games can raise awareness of health conditions, but they also learned about each other. One group member reflected *that ‘*everybody has some form of health issues and that you are not always aware of them’. They felt it was ‘amazing to work together to create this game’.

From observations of group dynamics from lead researchers, the group engaged enthusiastically with the game, and the discussions that were had were rich, deep and meaningful, facilitating a sense of understanding of each other. One community member commented in the feedback survey ‘I like the purple card game so you can explain your emotions’.

Working together to co‐design this game had a direct impact on our community partners and members. Several community members have continued to be involved in future grant applications. We left copies of the game at each Family Hub, and the community partners have both reflected on how the game continued to be used outside of the co‐design events. The game has also been used as a teaching resource for both students and academic staff to learn more about planning and delivering creative PPI events. Learnings have also been shared in public blogs. Finally, the game has been used in other research projects to encourage conversations about health, including with autistic adults. Future directions for this game include funding applications for further development of the game within different contexts, such as mental health and chronic pain. These examples of next steps emphasise that the co‐design approach generated value beyond the specific output (e.g., the game itself).

Overall, we were able to successfully use a combined participatory Listening Café and games‐based co‐design approach for public involvement with community members from underserved groups that enabled conversations about the challenges of living with health conditions. Through the co‐design process, community members felt that they had a voice. This game has a legacy far beyond this project in continuing conversations.

### 3.3 Lessons Learned From Combined Participatory Listening Café and Games‐Based Co‐Design Approach for Public Involvement

The lead researcher (Sian) and community partners (Sarah and Julie) engaged in written reflections. These are presented here as lessons learned, with quotes from community partners italicised for clarity, illustrating common ideas. The lessons learned were:

– Meaningful engagement
– Breaking down barriers to research involvement
– Achieving power balance and building relationships
– Cultural considerations

### 3.4 Meaningful Engagement

Sarah felt that research is often ‘completed by those who are quite removed from the subject matter, from those who are living day to day’. She felt that this project was different as it ‘comes from lived experiences which make it more relatable’. She enjoyed designing the game with community members, engaging in discussions about what games people enjoy and what the group felt was important so that it could ‘reach the widest audience and benefit many people’.

Julie reflected that ‘the opportunity to take part in this research was personal’ leaving her ‘feeling privileged at being a part of this research and the valued contribution we had given as a team and individuals’. She felt that ‘many of the group had not shared the details of their lives’ with each other before being involved in this project. This led to a positive outcome of ‘increased understanding’ of their lived experiences and ‘increased support and listening’. From being involved, she feels a greater empathy for others and has adapted her own leadership style as a result.

Sarah further reflected on her positive experience working with the artist who was ‘able to ask the right questions’. Sarah felt that the final design was ‘exactly how the group discussed, down to the colour scheme and pictures’. This gave everyone a ‘sense of achievement’. Sarah commented, ‘I personally found it very emotional knowing that I was part of such a great project and seeing the end result actually made me cry because I knew that the game would have so many positives.’

### 3.5 Breaking Down Barriers to Research Involvement

Julie had to navigate how to introduce the idea of PPI and encourage involvement from her community members. Julie requested community members to join in and negotiated about individuals ‘giving up their time’ to be involved. She used her leadership skills and facilitated a sense of being ‘all in this together’, focusing on the benefits of involvement in bringing the group closer together and building skills as a unit. As a result, her community members agreed and saw the benefit in bringing together their diverse backgrounds, skills and ethnicities.

Despite this, Julie reflected that ‘many of the group were feeling nervous of the unknown’. Sarah also mentioned this, with the idea of a game sounding somewhat ‘different’ and community members feeling unsure if they wanted to be involved. Sian reflects that using creative methods is a careful balance between something being new, exciting and meaningful versus scary, and not fully understanding what the purpose of the approach is. This comes down to clear communication and strong relationship foundations with key community partners who can help to champion the research.

Julie felt that the game ‘Me: Inside and Out’ is a useful game to support disclosure of feelings related to wellbeing, but also to enable inclusion, especially for people who are ‘quieter’. The cards gave them a focus and a voice. Sarah felt that this was an ‘opportunity to be part of something bigger and help break down barriers not just with discussions around long‐term health conditions but also the stigma around research and a whole different approach so that moving forward, research can be more accessible to the people that are meant to be the ones who benefit from the research in the future’. Sarah also said that this project has enabled her to ‘have conversations with individuals who are now keen to be involved in future research projects as the format and the outcome is appealing’.

### 3.6 Achieving Power Balance and Building Relationships

Sarah reflected on the sessions and how ‘when they flow easily, it enables everyone to be equal, which I felt this project was’ even though ‘at times there was a need for discussions to be steered, it was achieved naturally’. Sarah said that ‘in that room, we were all equal’. Sian reflects on the challenges of achieving this power balance. ‘As a researcher, you have a specific question or topic in mind. However, I wanted to remain open and allow our community members to shape exactly what this game would be and what it would ultimately mean to them. I am glad that we managed to achieve this in a way where everyone felt valued.’ Julie was ‘in awe of the trust built in such a small time’. Sian also felt that the co‐design process (and playing the game) allowed a platform for sharing experiences and creating a sense of ‘togetherness’.

### 3.7 Cultural Considerations

Julie felt that ‘the cultural diversity of the community group reflected how the caring of relatives with long term health needs was a big part of family life, including those in the UK and overseas’. This cultural diversity led to a discussion that was ‘rich and informative’.

With the Listening Café involving food, Julie felt ‘lunch was great but what we didn't factor in was it was Ramadan and a number of the team were fasting’. They were happy for us to carry on sharing lunch, as they said they were used to fasting, whilst others do not, and it was part of their faith and community to show this discipline. As a team, we reflected that we should give community members the choice of attending during Ramadan, allowing them to engage with events and their faith on their own terms. Sian also reflected that our planning team could have been more diverse, which might have helped consider these cultural issues sooner.

## 4 Discussion

### 4.1 Interpretation of Findings

This paper demonstrates that the combination of participatory Listening Cafés and a games co‐design approach for public involvement can engage underserved communities in meaningful conversations about their health. The ‘Me: Inside and Out’ card game facilitated emotionally rich, personal conversations that traditional PPI methods often fail to elicit. Community members reported feeling heard and valued, with the process fostering a sense of togetherness and mutual understanding. These findings align with prior work on the value of games in health communication [23, 25] but extend the literature by showing how games can be co‐created with communities using participatory Listening Café methods to foster engagement [45].

We found that creative co‐design methods, such as games, can effectively engage underserved communities in conversations about health, helping to overcome structural and cultural barriers that often limit participation. Creative methods, particularly games, can democratise research spaces by reducing power imbalances and making participation enjoyable and accessible. Community members described feeling ‘equal’ in the room, with the game acting as a leveller that encouraged quieter voices to contribute. This echoes Langley et al.'s (2018) concept of ‘collective making’ as a vehicle for knowledge mobilisation [15] and supports emerging evidence that games can foster emotional safety and cultural appropriateness [26, 27]. The emotional responses from community partners (such as pride, joy and even tears) highlight the profound personal impact of being genuinely involved in research.

**Figure 3:**
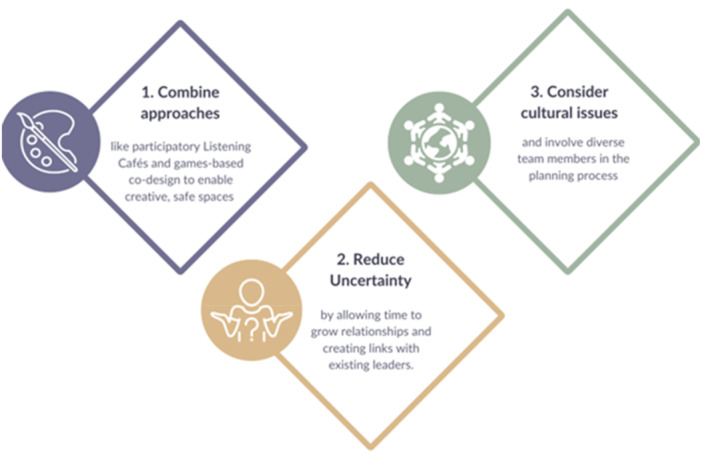
Lessons learned from the creative co‐design PPI approach.

The co‐design process revealed several critical enablers of success, with our lessons learned drawn together into an infographic, presented in Figure 3. First, the combined use of the games‐based approach and Listening Café model enabled a creative, safe and informal space for dialogue (see 1 in Figure 3). Hosting sessions in community‐led spaces and sharing food fostered psychological safety and openness. Furthermore, the collaborative development of the ‘Me: Inside and Out’ card game not only enhanced mutual understanding of lived experiences but also produced a reusable resource that continues to support public involvement in other research and educational contexts. For contexts where this specific game would not be appropriate, this paper presents an overall methodology for a combined participatory Listening Café and games‐based co‐design approach. Second, shared ownership of the game's content and taking time to build trust with community leaders contributed towards reduced feelings of uncertainty, particularly within the context of the novel combined Listening Café and games‐based approach for public involvement (see 2 in Figure 3). Third, flexibility and responsiveness were essential. The team adapted to cultural and religious contexts (e.g. Ramadan), demonstrating the importance of reflexivity and inclusive planning (see 3 in Figure 3). These findings reinforce the principles of Critical Participatory Action Research [14] and extend the NIHR INCLUDE framework by offering a replicable model for co‐designing games with underserved groups.

### 4.2 Strengths and Limitations

This is one of the first UK‐based publications to report on the co‐design of a PPI game with underserved communities to support conversations about health. Another strength is the methodological contribution, offering a practical, replicable model for inclusive, creative games co‐design using the Listening Café approach. Finally, this paper reinforces the value of co‐design and community engagement, contributing towards the growing literature on participatory design in health research.

To strengthen this work, the game should be tested with other groups to evaluate its value in other contexts. To improve future evaluations, we acknowledge the value of using validated tools such as the PIRIT (Public Involvement Impact Assessment Framework), which offers a structured approach to capturing and comparing PPIE outcomes [46]. Incorporating such tools could support more rigorous assessment of impact, including dimensions like feasibility, acceptability and sustainability.

### 4.3 Implications for Practice, Policy and Future Directions

This project offers a replicable model for engaging underserved communities in research through a combined methodological approach involving both Listening Cafés and games‐based co‐design for public involvement. It highlights the importance of flexibility, cultural awareness and relationship‐building in PPI. Policymakers and funders have a crucial role to play in supporting researchers to use similar approaches that prioritise inclusion and co‐production, particularly in involving underserved groups in innovative participatory approaches to shape the direction of research.

The game designed in this project (Me: Inside and Out) has since been used in teaching, further research, and community settings, demonstrating its adaptability and ongoing impact [47].

## 5 Conclusion (Summary of Key Messages and Future Directions)

This paper demonstrates how a combined participatory Listening Café and games‐based co‐design approach to public involvement (leading to the creation of games like Me: Inside and Out’) can meaningfully engage underserved communities in health research. By working in trusted, informal settings and sharing ownership of the process, community partners and members felt heard and connected with each other. Our lessons learned highlight the value of culturally sensitive, relationship‐driven approaches in breaking down barriers to involvement. Researchers can adopt and adapt this combined methodological approach for public involvement to create more inclusive, impactful research that reflects the voices of those it aims to serve. Future work should continue to explore how culturally responsive, relationship‐centred and creative games‐based approaches can shape more equitable and impactful research.

## Author Contributions

**Sian Holt:** conceptualization, methodology, formal analysis, investigation, resources, writing – original draft, visualization, supervision, project administration, funding acquisition. **Sarah Newman:** formal analysis, resources, writing – review and editing. **Julie Powell:** formal analysis, resources, writing – review and editing. **Kate Henaghan‑Sykes:** methodology, investigation, resources, writing – review and editing, project administration. **Sonia Newman:** methodology, investigation, resources, writing – review and editing, project administration. **Miriam Santer:** methodology, writing – review and editing. **Leanne Morrison:** methodology, writing – review and editing.

## Ethics Statement

This project was involvement rather than participation. Therefore, formal ethical review was not required, although ethical practice was adhered to throughout.

## Consent

Our community partners gave permission for their own words to be used to reflect upon their experiences.

## Conflicts of Interest

The authors declare no conflicts of interest.

## Data Availability

Data sharing not applicable to this article as no datasets were generated or analysed during the current study.

## Appendix A GRIPP‐2

**Table:**

Section/Item	Description	Status
Section 1: Abstract	1a. Aim	Yes—background
	1b. Methods	Yes—design
	1c. Results	Yes—results
	1d. Conclusions	Yes—conclusion
	1e. Keywords	Yes
Section 2: Background	2a. Definition of PPI	Yes—Pg 5
	2b. Theoretical underpinnings	Yes—Pg 8
	2c. Concepts and theory development	n/a
Section 3: Aims	3. Aim of the paper	Yes—Pg 6
Section 4: Methods	4a. Design of PPI	Yes—Pg 8–10
	4b. People involved	Yes—Pg 8–10
	4c. Stages of involvement	Yes—Pg 8–10
	4d. Level/nature of involvement	Yes—Pg 8–10
Section 5: Measurement of PPI Impact	5a. Qualitative evidence	Yes—Pg 10
	5b. Quantitative evidence	n/a
	5c. Robustness of measure	Reflected on in strengths/limitations
Section 6: Economic Assessment	6. Economic assessment	n/a
Section 7: Study Results	7a. Outcomes of PPI	Yes—Pg 11–14
	7b. Impacts of PPI	Yes—Pg 11–14
	7c. Contextual factors	Yes—Pg 11–14
	7d. Process factors	Yes—Pg 11–14
	7ei/ii. Theory development	n/a
	7f. Measurement tools	Appendix
	7g. Economic impact	n/a
Section 8: Discussion and Conclusions	8a. Overall influence of PPI	Yes—Pg 16–18
	8b. Contribution to knowledge	Yes—Pg 17–18
	8c. Definition of PPI	In introduction
	8d. Theoretical contribution	Yes—Pg 16–18
	8e. Contextual influence	Yes—Pg 16–18
	8f. Process influence	Yes—Pg 16–18
	8g. Measurement of impact	Yes—Pg 16–18
	8h. Economic aspects	n/a
	8i. Critical reflection	Yes—Pg 16–18

## Appendix B Example Documents Used

**Outline of Events**

**Table:**

Session no.	Planned activities
Session 1 NOTE: Bring post‐it notes, example games	Introductory session aimed at getting to know the group Aim of the session is priority setting. What do they think is most important? Order of events 1. Everyone arrives, Sonia introduces the session and researcher. Kate does an icebreaker, then encourages people to get some food 2. A comfort break around half‐way 3. The researcher introduces the project (video to support this: https://www.youtube.com/watch?v=qujfXz9LlLc) a. Why are we doing this/What are we hoping to achieve together? i. Living with health conditions is challenging and can impact day‐to‐day life in many ways. [video: https://www.youtube.com/watch?v=qujfXz9LlLc] We'd love to hear your voices and your stories about your health and day‐to‐day life, to understand how we can design research and support to help you and others in the future. b. Why a board game? What is the purpose? i. People of all ages play games – often bringing people together. Talking about challenges in our health can be difficult and board games can be a fun way to do this. We want to design a game together to help have these challenging conversations with other groups of people like yourself, and have their voices heard too. c. Explain how we hope this will make a difference for people living with MLTCs 4. Conversations/informal chat whilst doing some crafts Conversation starters (using prompt cards): ◦ Does your health make your day‐to‐day life more challenging? How? ◦ How do you have conversations about your health with other people? What makes this easier/difficult? ◦ What do you wish people understood about your health? ◦ What makes a game feel fun? Questions to interweave throughout to evaluate our progress (no prompt cards) ◦ Getting involved in research ▪ How did you find out about the Listening Café? What made you want to get involved? ▪ Do you normally get involved in research? ▪ Does this Listening Café feel similar or different to other things you've been involved in? ▪ What are the challenges for you to get involved in research? ◦ Co‐production ▪ How well do you think we are working together? ▪ How did we create something positive together? NOTE: Bring post‐it notes, example games
Session 2 NOTE: Bring post‐it notes, example games	Creative conversations about living with health conditions Aim of the session is to work together to discuss the challenges of living with MLTC, what makes a game fun, what would make this game worth playing and decide what is most important to include Order of events 1. Everyone arrives. Sonia welcomes and Kate does the icebreaker, then encourages people to have food 2. Sian brief recap then chat over food about challenges of living with MLTCs a. Everyone writes or draws something that they find challenging day‐to‐day with their health (post‐it notes) b. Gather up the post‐it notes and, as a group, stick them and discuss them. Maybe group them together. 3. Comfort break around halfway 4. Showing examples of games (health‐specific games and general games). Mind mapping ideas. a. What makes a game fun? b. Who do you play games with? c. If a game could help you with one thing about your health, what would it be?
Session 3 NOTE: Bring post‐it notes, example games	Continuing creative conversations about living with health conditions Aim of the session is to see what the visual artist has designed based on our last session, discussing what we think of the game and how we can improve it. Order of events 1. Everyone arrives. Sonia welcomes and Kate does the icebreaker, then encourages people to have food 2. Sian brief recap, then chat over food about the initial designs – visual artist and researcher explaining how the groups ideas have been translated into a game. a. What do we think? b. Does this capture the challenges? c. Would you play it? Who with? d. Do you think this game would help with the most important things you identified last time? 3. Comfort break around halfway 4. Continuing chats about the initial designs a. Writing down ideas on post‐it notes
Session 4 NOTE: Bring post‐it notes	Sharing the board game we have created together Aim of the session is to play the game and share conversations about the experience Order of events 1. Everyone arrives. Sonia welcomes and Kate does the icebreaker, then encourages people to have food 2. Sian brief recap, then show the group what they have created a. Breaking off into smaller groups to play the game 3. Comfort break around halfway 4. Group discussion on the experience of playing the game (post it note activity) a. What did you think? Was it fun? b. Does it capture the challenges? c. Would you play it? Who with? d. Do you think this game would help with the most important things you identified when we last met?
